# The incidence, risk factors and outcomes of early bloodstream infection in patients with malignant hematologic disease after unrelated cord blood transplantation: a retrospective study

**DOI:** 10.1186/s12879-018-3575-x

**Published:** 2018-12-13

**Authors:** Jing Ge, Tingting Yang, Lei Zhang, Xuhan Zhang, Xiaoyu Zhu, Baolin Tang, Xiang Wan, Juan Tong, Kaidi Song, Wen Yao, Guangyu Sun, Zimin Sun, Huilan Liu

**Affiliations:** 1 Department of Hematology of Anhui Provincial Hospital, Anhui Medical University, 17 Lujiang Road, Hefei, 230001 Anhui China; 2 Department of Hematology of Anhui Provincial Hospital, the First Affiliated Hospital of University of Science and Technology of China, Hefei, 230001 Anhui China

**Keywords:** Bloodstream infection, Cord blood transplantation, Myeloablative conditioning, Survival

## Abstract

**Background:**

Bloodstream infection (BSI) is one of the major causes of morbidity and mortality for patients undergoing hematopoietic stem cell transplantation (HSCT). The unrelated cord blood transplantation (UCBT) can provided opportunities for patients without suitable donors for bone marrow transplantation (BMT) and peripheral blood stem cell transplantation (PBSCT), while few studies have addressed BSI after UCBT. The aim of this study was to analyse the incidence and risk factors of BSI, causative organisms, microbial resistance, and its impact on the clinical outcomes and survival of patients.

**Methods:**

There are 336 patients, were divided into two groups depending on whether developing BSI. Demographic characteristics, laboratory data, and clinical outcome were compared between different groups. The risk factors of BSI was examined using logistic regression and the survival was examined using the Kaplan-Meier method and log-rank test.

**Results:**

Ninety-two patients (27.4%) developed early BSI with 101 pathogenic bacteria isolated, and the median day of developing initial BSI was 4.5 d. Gram-negative bacteria were the most common isolate (60, 59.4%), followed by Gram-positive bacteria (40, 39.6%) and fungi (1, 1.0%). Thirty-seven (36.6%) isolates were documented as having multiple drug resistance (MDR). Myeloid malignancies, conditioning regimens including total body irradiation (TBI), and prolonged neutropenia were identified as the independent risk factors for early BSI. The 3-year OS was 59.9% versus 69.2% in the BSI group and no-BSI group (*P* = 0.0574), respectively. The 3-year OS of the MDR group was significantly lower than that of the non-BSI group (51.1% versus 69.2%, *p* = 0.013).

**Conclusions:**

Our data indicate that the incidence of early BSI after UCBT was high, especially in patients with myeloid disease and a conditioning regimen including TBI and prolonged neutropenia. Early BSI with MDR after UCBT had a negative impact on long-term survival.

## Background

Infection is a common complication after hematopoietic stem cell transplantation (HSCT), and bloodstream infection (BSI) is one of the major causes of morbidity and mortality for patients undergoing HSCT [1]. In the past few years, the development of unrelated cord blood transplantation (UCBT) has provided opportunities for patients without suitable donors for bone marrow transplantation (BMT) and peripheral blood stem cell transplantation (PBSCT). However, due to the characteristics of UCBT, such as prolonged neutrophil recovery and delayed immune reconstitution, the incidence of infection after UCBT has increased significantly [2, 3]. Although the number of patients undergoing UCBT has increased rapidly, few studies have addressed BSI after UCBT. To the best of our knowledge, a small number of studies mostly from Japan and Spain with large sample sizes have evaluated the early incidence of BSI after cord blood transplantation [1, 4–8].

In this paper, we reported the results of our analysis of early BSI in 336 patients undergoing UCBT at a single institution. The aim of this study was to analyse the incidence and risk factors of BSI, causative organisms, microbial resistance, and its impact on the clinical outcomes and survival of patients.

## Methods

### Patients

Between January 2012 and January 2017, 361 consecutive patients with malignant hematologic disease undergone single-unit UCBT following a myeloablative conditioning regimen in our transplantation centre. These patients did not have suitable related or unrelated bone marrow transplantation donors. Among the 361 patients, 25 suffered from primary engraft failure and were excluded from this analysis. Therefore, the final data analysis included 336 cases.

### Transplantation procedures

All patients received a myeloablative conditioning regimen including or not including total body irradiation (TBI). All patients were treated with cyclosporine A (CsA) (Novartis, Stein, Switzerland) and mycophenolate mofetil (MMF) (Roche, Basel, Switzerland) for the purpose of GVHD prophylaxis. CsA was administered from day − 1 at 3 mg/(kg.d) intravenously, then oral CsA at a dose ratio of 1:2 when oral intake could be tolerated with a trough level of 150–250 ng/mL. CsA was discontinued on day 180 or before if no GVHD occurred. From day + 1 to day + 28, patients took MMF orally three times a day to the total dose of 30 mg/(kg.d). The rapidity of tapering was based on the presence or absence of GVHD, infectious disease, and relapse risk. Infection prophylaxis consisted of oral Itraconazole, oral cefprozil and intravenous acyclovir. Ganciclovir or foscarnet was applied as a pre-emptive CMV therapy. Serum levels of C-reactive protein (CRP) were measured at least three times a week or at times of fever. During the pre-engraftment period, patients who experienced a neutropenic fever would be treated with broad-spectrum antibiotic. In the process of neutropenic fever (with body temperature ≥ 38 °C), patients received an empirical antibiotic regimen consisted of imipenem/cilastatin and aminoglycoside (amikacin), which was later modified in accordance with their clinical condition and the results of microbial culture. In the event that the fever continued for 48 h and/or the clinical symptoms got worse, patients would receive intravenous vancomycin; in the event that the fever failed to respond to these regimens by day 5, patients would receive the empirical antifungal treatment with intravenous liposomal amphotericin B or caspofungin. At the onset of fever or in the presence of any clinical symptom compatible with an infection, 2 sets of blood cultures were drawn from the peripheral vein and central catheter, and identification and an antimicrobial susceptibility test were performed. Mueller. Hinton Agar containing the 50 g/L off-fiber blood of Sheep was an antimicrobial-sensitive medium. Based on the protocol of drug susceptibility test recommended by the Clinical and Laboratory Standards Institute (CLSI), the results were analyzed by the MicroScan Walkaway 96 PLUS (U.S. Siemens) and Vitek 2 compact (French bioMerieux). Quality control strains were provided by the Ministry of Health Clinical Testing Center including: *Escherichia coli* ATCC 25922, Pseudomonas aeruginosa ATCC 27853, *Klebsiella pneumoniae* ATCC700603, *Staphylococcus aureus* ATCC 25923, Streptococcus pneumoniae ATCC 49619.

### Definitions

Neutrophil recovery was the first day of three consecutive days during which the absolute neutrophil count (ANC) exceeded 0.5 × 10^9^/L. Neutropenia and severe neutropenia were defined as an absolute granulocyte count < 0.5 and < 0.1 × 10^9^/L, respectively. Patients who survived for more than 28 days after transplantation but failed to achieve neutrophil recovery and with < 5% cells of donor origin were regarded as having primary engraft failure. BSI was defined as the isolation of bacteria or fungi from any blood culture in the context of fever or other clinical signs consistent with infection. For coagulase-negative Staphylococci, Streptococcus viridans and other common commensal at least 2 blood cultures were required to be positive [9, 10]. BSI was considered to be polymicrobial when more than 2 pathogens were isolated in the same blood culture or in at least 2 separate blood cultures in a time period of 72 h [9]. Multiple drug resistance (MDR) was defined as acquired non-susceptibility to at least 1 usually active agent in 3 or more antimicrobial categories following the criteria proposed by Magiorakos et al. [11]. BSI occurred in neutropenia (absolute granulocyte count < 0.5 × 10^9^/L) during the UCBT was identified as having early BSI. Pathogens with intermediate susceptibility or resistance were considered resistant. In this research, the prolonged neutropenia was identified by more than the observed median time of neutrophil recovery in all patients. Overall survival (OS) was defined as the time from UCBT to death or to the last observation. Disease-free survival (DFS) was defined as the time from UCBT to relapse, death or the last observation.

### Data collection

Data on the patients’ characteristics, transplantation procedures, and infectious complications were collected once patients entered the laminar flow ward and were then stored in a computerized database. In cases of inconsistent or missing data, clinical charts were reviewed. A follow-up study on each transplant case was performed annually to update the data on relapse, survival, and complications. The final data set used for analysis was established on October 1, 2017.

### Statistical analysis

Categorical variables were compared with the chi-squared test or Fisher’s exact test (both 2-sided) and the Mann–Whitney U test for continuous variables. Continuous variables are expressed as the median and range. The cut-off value for continuous variables was selected based on the observed median (except for age, which was divided into < 14 vs. 14 years and older). Various clinical factors were evaluated as potential risk factors for BSI in univariate and multivariate analyses combined with the logistic regression model. Factors found to be significant (*P* < 0.05) or marginally significant (*P* < 0.20) in univariate analysis were included in multivariate analysis using a forward stepwise method to explore the independent effects of different variables on the incidence of BSI.

The OS of different groups was determined using the Kaplan-Meier method and log-rank test and the DFS was determined using survival analysis containing competing event. A *P* value < 0.05 was considered statistically significant. All statistical analyses were carried out using SPSS 18.0 (SPSS, Inc., Chicago, IL, USA) and R version 3.0.1 (The CRAN project).

## Results

### Patients’ characteristics

Table 1 shows the basic clinic characteristics of all patients included in the final analysis. Among the 336 patients, 215 patients were males and 223 were children. The median weight of patients was 37 kg (range, 10 to 82). The diagnoses included ALL (52.7%), AML (32.8%), CML (4.8%) and MDS (6.5%), and others (3.8%). A total of 257 patients (75.6%) were in complete remission (CR) or the chronic phase, whereas the remaining patients were in no remission (NR) or the advanced phase. Among HLA matching (low-resolution), 113 patients (33.6%) were 4/6 matched, 168 patients (50.0%) were 5/6 matched, and 55 patients (16.4%) were fully matched. All patients received a myeloablative conditioning regimen, 109 (32.4%) received total body irradiation (TBI), and 227 (67.6%) did not receive TBI. The median number of total nucleated cells (TNC) and CD34-positive cells in infused cord blood was 4.17 × 10^7^/kg (range, 1.71 to 13.90) and 2.32 × 10^5^/kg (range, 0.40 to 10.55), respectively.

**Table 1:** Clinical characteristics of patients

	Values (336)
Gender [No. (%)]	
Male	215(64.0)
Female	121(36.0)
Age [years]	
< =14	223(66.4)
> 14	113(33.6)
Median (range)	12 (2–55)
Body weight(kg)median(range)	37(10–82)
Disease [No. (%)]	
AML	110(32.7)
ALL	177(52.7)
CML	16(4.8)
MDS	22(6.5)
Others	11(3.3)
State of disease [No. (%)]	
CR or Chronic phase	257(76.5)
NR or Advanced phase	79(23.5)
HLA match (low-resolution) [No. (%)]	
4/6	113(33.6)
5/6	168(50.0)
6/6	55(16.4)
Conditioning regimen [No. (%)]	
Including TBI	109(32.4)
Not including TBI	227(67.6)
Total nucleated-cell dose, 10^7^/kg median(range)	4.17(1.71–13.90)
Total CD34+ cell dose, 10^5^/kg median(range)	2.32(0.40–10.55)

*AML* acute myeloid leukaemia, *ALL* acute lymphoblastic leukaemia, *CML* chronic myeloid leukaemia, *MDS* myelodysplastic syndrome, *HLA* human leukocyte antigen, *TBI* total body irradiation, *CR* complete remission, *NR* no remission

### Incidence and aetiology of early BSI

In all patients, 92 patients (27.4%) were documented to have early BSI, 5 patients with twice the occurrence of BSI, and 4 patients with two types of pathogenic bacteria in the same blood culture. Finally, 101 pathogenic bacteria were isolated. The median day of initial BSI was 4.5 d (range, − 3 d to + 36 d). Within + 7 d, the cumulative frequency of BSI was 84 times, accounting for 91.3% of cases.

Overall, there were 101 isolates over 97 episodes of BSI. Table 2 shows the specific isolated pathogens of BSI after UCBT. Gram-negative bacteria accounted for 60 isolates (59.4%), Gram-positive bacteria for 40 isolates (39.6%), and documented fungi 1 isolate (*Candida albicans*). The predominant Gram-negative bacteria in descending order were as follows: *Escherichia coli* (39 isolates), *Klebsiella pneumoniae* (9 isolates), and Pseudomonas aeruginosa (6 isolates). The predominant Gram-positive bacteria were Streptococcus viridans (10 isolates), *Staphylococcus epidermidis* (9 isolates), Enterococcus faecium (7 isolates), and Streptococcus mitis (7 isolates). In all isolated pathogens, the number of *Escherichia coli* was the most, while ESBL-producing *E. coli* represented 89.7%. In the case of *Klebsiella pneumoniae*, 6 isolates (66.7%) produced ESBL. No MRSA was documented in our study.

**Table 2:** Isolated pathogens of BSI after UCBT

Pathogens (n)	Isolates, n (%)
Gram-negative bacteria (60)	
*Escherichia coli*	39(38.6)
*Klebsiella pneumoniae*	9(8.9)
Pseudomonas aeruginosa	6(5.9)
Sphingomonas paucimobilis	1(1.0)
Enterobacter cloacae	1(1.0)
Microbacterium flavescens	2(2.0)
Citrobacter freundii	1(1.0)
Acinetobacter baumanii	1(1.0)
Gram-positive bacteria(40)	
Streptococcus viridans	10(10.0)
*Staphylococcus epidermidis*	9(8.9)
Enterococcus faecium	7(6.9)
Streptococcus mitis	7(6.9)
Streptococcus pluranimalium	3(3.0)
Gordona spp.	1(1.0)
Staphylococcus human	1(1.0)
Staphylococcus ear	1(1.0)
Gemella spp.	1(1.0)
*Candida albicans*(1)	1(1.0)

*BSI* bloodstream infection

As for antibiotic susceptibility, considering the category and quantity of the isolated bacteria, we selected *Escherichia coli*, *Klebsiella pneumoniae*, and Pseudomonas aeruginosa among Gram-negative bacteria and Streptococci, coagulase-negative Staphylococcus (CoNS), and Enterococcus faecium among Gram-positive bacteria to analyse their resistance to common antibacterial drugs. The results are shown in Tables 3 and 4. We found that Gram-negative bacteria except for *Klebsiella pneumoniae* presented a low rate of resistance to imipenem/cilastatin and amikacin and a relatively high rate of resistance to penicillin, third- or fourth-generation cephalosporins and quinolones. 33.3% *Klebsiella pneumoniae* were resistance to imipenem/cilastatin. Although Gram-positive bacteria possessed a rather large number of streptococci and CoNS, they were mostly opportunistic pathogens with a low resistance rate. Most Enterococcus faecium specimens were resistant to penicillin and quinolones, while they were 100.0% sensitive to vancomycin, linezolid and tigecycline. In addition, we recorded 37 isolates (36.6%) of MDR. MDR were all Gram-negative bacteria, and five of the nine isolates of Klebsiella pneumonia (55.6%) and 32 of the 39 isolates of *Escherichia coli* (82.1%) were MDR. In this set of data, we found that *Escherichia coli* presented low resistance rates to imipenem/cilastatin, amikacin, and piperacillin/tazobactam, which were 5.1, 12.8, and 15.4%, respectively. Only 2 strains of carbopenem-resistant *Escherichia coli* were discovered, but they were sensitive to amikacin.

**Table 3:** Antibiotic resistance in Gram-negative isolates

Antibiotics	Escherichia coli,% (*n* = 39)	Klebsiella pneumoniae, % (*n* = 9)	Pseudomonas aeruginosa, % (*n* = 6)	MDR bacteria, % (*n* = 37)
Amikacin	12.8	44.4	0	18.9
Ampicillin	100	100	33.3	100
Aztreonam	89.7	88.9	50	91.9
Ceftazidine	76.9	100	0	100
Ciprofloxacin	69.2	66.7	0	91.9
Ceftriaxone	100	100	33.3	100
Cefepime	82.1	100	0	100
Gentamicin	74.4	77.8	0	81.1
Imipenem	5.1	33.3	0	13.5
Levofloxacin	64.1	55.6	0	75.7
Ampicillin-Sulbactam	97.4	100	33.3	100
Compound Sulfamethoxazole	87.2	100	33.3	100
Piperacillin	15.4	0	0	16.2

**Table 4:** Antibiotic resistance in Gram-positive isolates

Antibiotics	Streptococcus, % (*n* = 20)	Coagulase-negative Staphylococcus, % (*n* = 11)	Enterococcus faecium, % (*n* = 7)
Ciprofloxacin	一	36.4	100
Erythromycin	55	72.7	100
Gentamicin	一	63.6	85.7
Linezolid amide	一	0	0
Levofloxacin	5	36.4	100
Penicillin	55	81.8	85.7
Dalfopristin	一	0	0
Streptomycin	一	一	57.1
Tetracyclines	一	36.4	28.6
Tigecycline	一	0	0
Vancomycin	0	0	0

一, Did not carry out drug resistance test

### Risk factors for early BSI after UCBT

Table 5 shows the differences in the clinical characteristics of 92 patients with BSI and 244 patients without BSI and the risk factors for developing early BSI according to univariate and multivariate analyses. In univariate analysis, the age with the best cut-off at 14 years (*p* = 0.037), disease (*p* < 0.001), conditioning regimen including or not including TBI (*p* = 0.001), state of disease (*p* = 0.016), number of TNC with a best cut-off at 4.17 × 10^7^/kg (*p* = 0.035), and time of neutrophil recovery with a best cut-off at 17d (p < 0.001) were statistically significant. And Total CD34+ cell dose with a best cut-off at 2.32 × 105/kg (*p* = 0.079) was marginally significant. Multivariate analysis showed that ALL compared with myeloid disease (OR = 0.611, 95%CI 0.343–1.089, *P* = 0.007), a conditioning regimen including TBI (OR = 1.755, 95%CI 1.008–3.057, *P* = 0.047), and prolonged neutropenia (>17d, OR = 1.117, 95%CI 1.056–1.118, *p* < 0.001) were statistically significant for early BSI after UCBT.

**Table 5:** Univariate and multivariate analyses of risk factors for the early BSI after UCBT

Factor	BSI (*n* = 92)	non-BSI (*n* = 244)	Univariate analysis	Multivariate analysis	
			*P*	OR (95% CI)	*P*
Gender			0.634		
Male	57	158			
Female	35	86			
Age [years]			0.037		0.164
<=14	53	170			
> 14	39	74			
Disease			< 0.001		0.007
AML	33	77			
ALL	34	143		0.611(0.343–1.089)	
CML	8	8		1.578(0.482–5.168)	
MDS	12	10		2.752(1.015–7.464)	
Others	5	6		2.870(0.769–10.710)	
Conditioning regimen			0.001	1.755(1.008–3.057)	0.047
Not including TBI	49	178			
Including TBI	43	66			
State of disease			0.016		0.640
CR or Chronic phase	62	195			
NR or Advanced phase	30	49			
HLA match (low-resolution)			0.75		
4/6	33	80			
5/6	46	122			
6/6	13	42			
Total nucleated-cell dose			0.035		0.940
< =4.17 × 10^7^/kg	55	114			
> 4.17 × 10^7^/kg	37	129			
Total CD34^+^ cell dose			0.079		0.977
< =2.32 × 10^5^/kg	54	117			
> 2.32 × 10^5^/kg	38	127			
Time of neutrophil recovery			< 0.001	1.117(1.056–1.118)	0.000
< =17d	34	150			
> 17d	58	94			

*OR* odds ratio, *CI* confidence interval

### Impact of early BSI on the clinical results

We also compared the differences in developing acute graft-versus-host disease (aGVHD), pre-engraftment syndrome (PES), and some other clinic indexes between the BSI group and non-BSI group. The incidence of acute GVHD (40.2% vs 41.0%, *p* > 0.05), PES (76.1% vs 79.1%, *p* > 0.05), and III-IV aGVHD (15.2% vs 12.7%, *p* > 0.05), the median length of time that patients stay in laminar flow ward (37d vs 42d, *p* > 0.05) showed no significant differences in the two groups. Some clinical indexes, such as the CRP maximum value (106 mg/L vs. 60.6 mg/L, *p* < 0.05), day of the CRP maximum value (7d vs. 8d, *p* < 0.05) and day of first fever (4d vs 5d, *p* < 0.05), were significantly different between the two groups.

### Impact of early BSI on survival

**Fig. 1:**
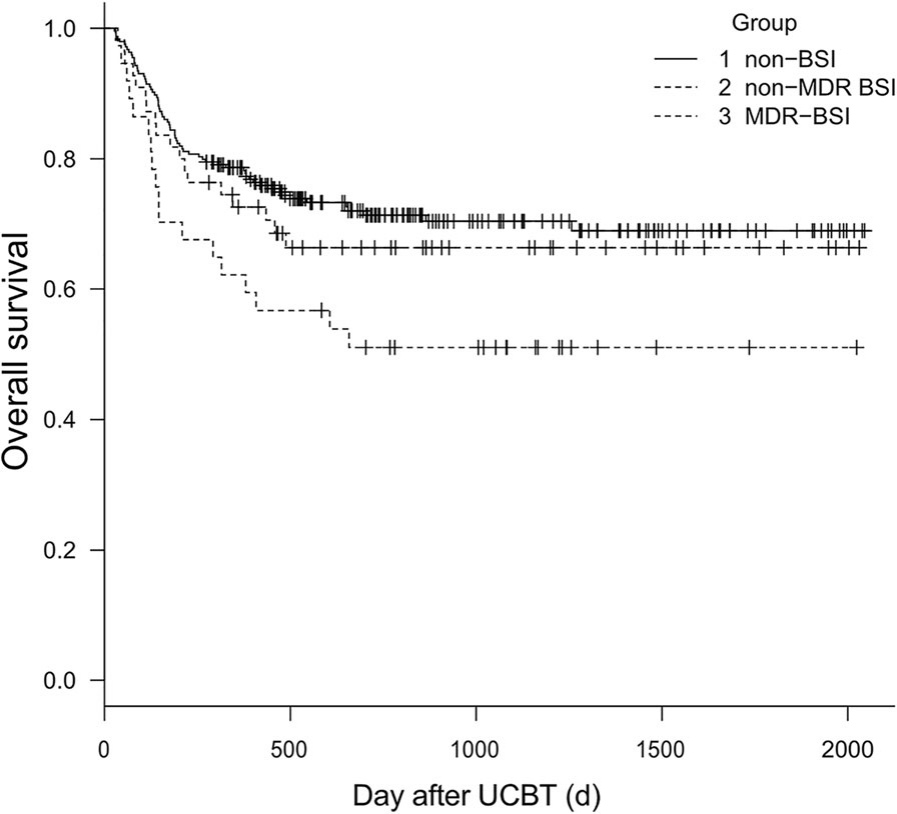
Kaplan--Meier estimate of OS for 336 patients without BSI, with MDR-BSI, and with non-MDR BSI after UCBT. The 3-year OS was 51.1% versus 69.2% (*p* = 0.013) in MDR group and non-BSI group

**Fig. 2:**
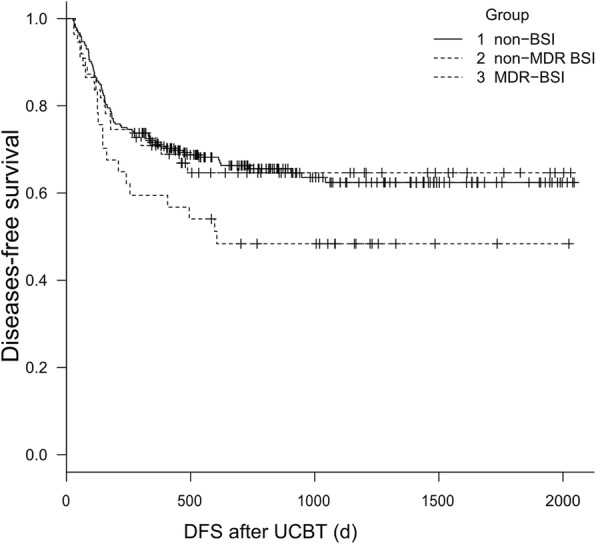
Kaplan--Meier estimate of DFS for 336 patients without BSI, with MDR-BSI, and with non-MDR BSI after UCBT. The 3-year DFS was 48.4% versus 62.4% (*p* = 0.067) in MRD group and without BSI group

The median follow-up of surviving patients was 584 d (range, 27 to 2045 d). First, we compared the differences in survival depending on whether they developed BSI after UCBT. The median day of OS in patients with BSI was 1304 d, while in patients without BSI, it was 1503 d (*p* = 0.055). The 3-year OS was 59.9% (95% CI, 0.489–0.692) versus 69.2% (95% CI, 0.622–0.752) in patients with BSI and without BSI, respectively (*P* = 0.0574), and the 3-year DFS was 57.7% (95% CI, 0.476–0.672) versus 62.4% (95%CI, 0.551–0.689) in these two groups, respectively (*P* = 0.257). Considering the influence of BSI with MDR on prognosis, we made a more detailed grouping. Figures 1 and 2 show the differences in OS and DFS in non-BSI, MDR-BSI, and non-MDR BSI group. The OS of MDR group was significantly lower than that of the non-BSI group (*p* = 0.012). The 3-year OS was 51.1% (95% CI, 0.341–0.658) versus 69.2% (95% CI, 0.622–0.752, *p* = 0.013) in the two groups, and the 3-year DFS was 48.4% (95% CI, 0.316–0.632) versus 62.4% (95% CI, 0.551–0.689, *p* = 0.067). The OS and DFS in the non-BSI group and non-MDR BSI group were not different. We also analysed the 100-day OS after UCBT between these three groups, and no difference was found. And considering the AML was identified as a independent risk factor for developing BSI in multivariate analysis, we did separate outcome analysis for AML patients, the results showed that there were 42 patients developing BSI in all 110 patients. The short-term and long-term survival were no significant difference in BSI group and non-BSI group. While, in MDR-BSI and non-BSI group, the long-term survival was significantly different. The 3-year OS was 50.0% (95%CI, 0.403–0.691) versus 79.4% (95% CI, 0.684–0.911, *p* = 0.006), and the 3-year DFS was 48.2% (95% CI, 0.382–0.603) versus 77.1% (95% CI, 0.695–0.883, *p* = 0.013) in these two groups.

## Discussion

Bloodstream infection remains a major complication following UCBT [1]. The incidence of early BSI after UCBT ranged from 11 to 35% in previous studies [1, 4, 5, 8, 12]. In our data, 27.4% of patients developed early BSI after UCBT. The different transplantation centres and sizes of samples led to a variation in the incidence of BSI. Compared with other transplantation types, developing BSI was more common in UCBT [13] because UCBT has unique and inherent immunological properties and peculiarities. Delayed neutrophil recovery and slow immune reconstruction after UCBT could be expected to contribute to severe BSI as neutrophils represent a major protective factor against infections [14]. Another contributing factor is that immune cells in cord blood are mostly naive and have low protective effects against infection [15].

The prevalence of pathogens with dominant Gram-negative bacteria found in this study was consistent with several related studies in China and some developing countries [1, 16, 17]. A study carried out by Peking University in 2010 reported that in 75 patients with BSI, the incidences of GNB, GPB, and fungi were 64.4, 30.1, and 5.5%, respectively [18]. Another Chinese study in 2015 reported the incidences of GNB, GPB, and fungi were 70.3, 26.9, and 2.8%, respectively, in patients after HSCT [19]. On the contrary, in developed countries, most published studies demonstrated that Gram-positive bacteria were more commonly found (50–80%) in patients with BSI after allo-HSCT, including UCBT [4–6, 20]. And the *Klebsiella pneumoniae* showed the high resistance to imipenem, it’s consistent with recent studies. The resistance mechanism may associated to the expression of CTX-M-15 caused by carbapenemases and the lack of porins on outer membrane of cells [21]. In our centre, quinolone was not conventionally used in gut decontamination before transplantation and methotrexate was not used for GVHD prophylaxis, which can reduce the damage to intestinal mucous membrane. And these strategies may result in the low incidence of BSI with GPB compared to GNB-BSI in this study. Our study reflected the local epidemiology in China, which was significantly different from that Europe, North American and Japan. It is meaningful to be aware of the epidemiology and drug resistance of bacteria and choose the most appropriate empirical antibacterial therapy.

In this study, we identified the disease and a conditioning regimen including TBI and prolonged neutropenia (>17d) as independent risk factors for early BSI after UCBT. In previous research, the prolonged neutropenia phase was described to be an independent risk factor for infection [22]. Sanz et al. [1] found early BSI to be a strong and independent predictor of neutrophil recovery as a time-dependent covariate, and the number of CD34-positive cells was a risk factor of BSI after UCBT. In another study, Gudiol et al. [8] found that the prolonged neutropenia phase, severe mucositis and CVC were risk factors of BSI in pre-engraftment after HSCT. In our data, patients with AML or CML had a high risk of developing early BSI compared with ALL, which can be explained by the fact that most ALL were children and commonly in CR when undergoing UCBT. Neutropenia was significantly prolonged in the BSI compared with the no-BSI groups. Considering that the time of initial BSI was 4.5 d and 91.3% of BSI occurred before + 7 d, the possibility that BSI delayed neutrophil recovery cannot be ruled out. The specific connection between early BSI and prolonged neutropenia requires further study. TBI in a conditioning regimen may aggravate damage to the intestinal mucosa, making BSI more likely to occur. The identification of risk factors for BSI may aid in efforts to reduce transplantation-related mortality. Patients with BSI often had earlier fever and higher CRP. This was consistent with other studies, which reported that CRP increased daily before confirmed BSI [23]. These changes were associated with the body’s inflammatory reaction due to BSI.

Regarding survival, we found that the 3-year OS of patients with BSI (59.9%) was lower than that of the patients without BSI (69.2%) with marginal significance (*p* = 0.0574), while the 3-year DFS in these two groups were not different. Moreover, patients developing MDR-BSI usually had a worse prognosis compared with patients without BSI. These findings emphasize the importance of preventing early BSI after UCBT to improve the prognosis of patients. Meanwhile, early BSI-related mortality did not increase in our data, which was inconsistent with other studies [4, 24]. Imipenem/cilastatin combined with amikacin, which had the best susceptibility to *Escherichia coli*, was the empiric antibacterial therapy used in our centre. Antibiotics were changed over time before we received the antimicrobial susceptibility test results, and imipenem/cilastatin was not used for more than 1 week to reduce the occurrence of carbopenem-resistant Enterobacteriaceae. These effective strategies for potential infection and support treatment may have contributed to the low mortality in the early phase after UCBT in patients with BSI. And the immune damage caused by MDR bacteria, depletion of the intestinal microbiota and secondary bone marrow suppression caused by the use of broad-spectrum antibiotics may lead to poor long-term survival in patients with MDR infection [25–27].

## Conclusions

we found that the incidence of early BSI in patients with malignant hematologic disease after UCBT was common, particularly in patients with myeloid disease and conditioning regimens including TBI. Patients who developed BSI, especially with the MDR, had worse survival. Understanding the epidemiology and antibiotic resistance of pathogens was of guiding significance for the selection of empirical antibiotics. Early diagnosis of BSI and prompt treatment with effective antibiotics are necessary for patients undergoing UCBT.

## Abbreviations

ANC: absolute neutrophil count
BMT: bone marrow transplantation
BSI: bloodstream infection
CoNS: coagulase-negative staphylococcus
CRP: C-reactive protein
CsA: cyclosporine A
DFS: disease-free survival
GVHD: graft-versus-host disease
HSCT: hematopoietic stem cell transplantation
MDR: multiple drug resistance
MMF: mycophenolate mofetil
OS: overall survival
PBSCT: peripheral blood stem cell transplantation
PES: pre-engraftment syndrome
TBI: total body irradiation
UCBT: unrelated cord blood transplantation
